# Effects of different energy levels in low-protein diet on liver lipid metabolism in the late-phase laying hens through the gut-liver axis

**DOI:** 10.1186/s40104-024-01055-y

**Published:** 2024-07-11

**Authors:** Hong Hu, Ying Huang, Anjian Li, Qianhui Mi, Kunping Wang, Liang Chen, Zelong Zhao, Qiang Zhang, Xi Bai, Hongbin Pan

**Affiliations:** 1 Yunnan Provincial Key Laboratory of Animal Nutrition and Feed Science, Faculty of Animal Science and Technology, Yunnan Agricultural University, Kunming, 650201 China; 2 College of Animal Science, Anhui Science and Technology University, Bengbu, 233000 China; 3 State Key Laboratory of Animal Nutrition, Institute of Animal Sciences, Chinese Academy of Agriculture Sciences, Beijing, 100193 China; 4 Shanghai BIOZERON Biotechnology Co., Ltd, Shanghai, 201800 China; 5 WOD Poultry Research Institute, Beijing, 100193 China

**Keywords:** Cecal microbiome, Energy/protein imbalance, Late-phase laying hens laying hens, Liver lipid metabolism, Low-protein diet, Multi-omics

## Abstract

**Background:**

The energy/protein imbalance in a low-protein diet induces lipid metabolism disorders in late-phase laying hens. Reducing energy levels in the low-protein diet to adjust the energy-to-protein ratio may improve fat deposition, but this also decreases the laying performance of hens. This study investigated the mechanism by which different energy levels in the low-protein diet influences liver lipid metabolism in late-phase laying hens through the enterohepatic axis to guide feed optimization and nutrition strategies. A total of 288 laying hens were randomly allocated to the normal-energy and normal-protein diet group (positive control: CK) or 1 of 3 groups: low-energy and low-protein diet (LL), normal-energy and low-protein diet (NL), and high-energy and low-protein diet (HL) groups. The energy-to-protein ratios of the CK, LL, NL, and HL diets were 0.67, 0.74, 0.77, and 0.80, respectively.

**Results:**

Compared with the CK group, egg quality deteriorated with increasing energy intake in late-phase laying hens fed low-protein diet. Hens fed LL, NL, and HL diets had significantly higher triglyceride, total cholesterol, acetyl-CoA carboxylase, and fatty acid synthase levels, but significantly lower hepatic lipase levels compared with the CK group. Liver transcriptome sequencing revealed that genes involved in fatty acid beta-oxidation (*ACOX1*, *HADHA*, *EHHADH*, and *ACAA1*) were downregulated, whereas genes related to fatty acid synthesis (*SCD*, *FASN*, and *ACACA*) were upregulated in LL group compared with the CK group. Comparison of the cecal microbiome showed that in hens fed an LL diet, *Lactobacillus* and *Desulfovibrio* were enriched, whereas riboflavin metabolism was suppressed. Cecal metabolites that were most significantly affected by the LL diet included several vitamins, such as riboflavin (vitamin B_2_), pantethine (vitamin B_5_ derivative), pyridoxine (vitamin B_6_), and 4-pyridoxic acid.

**Conclusion:**

A lipid metabolism disorder due to deficiencies of vitamin B_2_ and pantethine originating from the metabolism of the cecal microbiome may be the underlying reason for fat accumulation in the liver of late-phase laying hens fed an LL diet. Based on the present study, we propose that targeting vitamin B_2_ and pantethine (vitamin B_5_ derivative) might be an effective strategy for improving lipid metabolism in late-phase laying hens fed a low-protein diet.

**Supplementary Information:**

The online version contains supplementary material available at 10.1186/s40104-024-01055-y.

## Background

With an increase in living standards, the demand for food quality has also increased. Owing to the high nutritional value of eggs, laying hen breeding has become a rapidly expanding area in the poultry farming industry [1]. Although modern, highly intensive breeding methods for laying hens have improved breeding efficiency, convenience, and effectiveness, these methods also reduce the activity of laying hens, resulting in excessive energy intake that is converted into fat deposition in the liver, leading to fatty liver, stress, and increased mortality [2, 3]. The late-phase laying hens are particularly prone to lipid metabolism disorders after experiencing peak production, which may lead to fat accumulation in the liver, reduced egg production quality, increased mortality, and ultimately, economic losses [4].

A low-protein (LP) poultry diet is valuable due to several advantages including lower feed costs, reduced industry dependence on dietary crude protein, and decreased nitrogen and ammonia pollution [5–7]. Many studies have suggested that the energy/protein imbalanced low-protein diet enhances the risk of lipid metabolism disorders in late-phase laying hens and negatively impacts egg production rates, egg weight, and feed intake [8–10]. These effects may be due to unbalanced energy and protein levels in the diet [11]. Reducing energy levels in the LP diet to adjust the energy-to-protein ratio may improve fat deposition, but it also decreases the growth performance of chickens [12]. Feed nutrition optimization is an effective method for preventing the negative effects of LP diets [13]. However, it is difficult to develop functional feed additives that can improve lipid metabolism in the liver of late-phase laying hens. Therefore, there is an urgent need to investigate the underlying mechanisms of liver lipid metabolism in late-phase laying hens (LP diet with unbalanced energy/protein ratio) to better guide feed nutrition strategies.

Previous studies have indicated the important roles of the gut microbiota in fatty acid disease, which can affect the liver through the gut-liver axis [14]. The gut microbiota influences energy storage, lipid and choline metabolism, ethanol production, immune balance, and inflammation, all of which are relevant to the pathogenesis of lipid metabolism disorders [15]. Furthermore, changes in the gut microbiota composition have been associated with the progression of lipid metabolism disorders and the degree of fibrosis [16]. In laying hens, previous studies have shown that alterations in the cecal microbiota are closely linked to the severity of liver conditions, with changes in the abundance of specific bacterial groups associated with the progression of hepatic steatosis and lipid metabolism disorders [17, 18]. Previous research on lipid metabolism disorders in poultry has primarily focused on energy intake, and calcium deficiency [19, 20], whereas research related to protein intake is limited. Understanding the interactions between gut microbiota and liver health in laying hens fed an energy/protein imbalanced LP diet is crucial for developing effective strategies to prevent and manage lipid metabolism disorders. Therefore, this study aimed to combine multiple omics techniques (transcriptomics, 16S, and metabolomics) to comprehensively analyze the impact of the energy/protein imbalanced LP diet on liver lipid metabolism in late-phase laying hens through the gut-liver axis. The results of this study are expected to be of great significance in protecting laying hens from liver lipid metabolism disorders.

## Methods

### Hens and dietary treatment

A total of 288 laying hens (aged 57 weeks) of strain Peking Pink (YunLing GuangDa Yukou Poultry Co. Ltd., Yunnan, China) with similar body weight were randomly divided into the following 4 groups (6 replicates of 12 hens per group): the control (CK) group, fed a normal-energy and normal-protein diet (11.15 MJ/kg, 16.59% CP), and 3 LP groups: (1) the low-energy and low-protein (LL) diet group (10.73 MJ/kg, 14.50% CP), (2) the normal-energy and low-protein (NL) diet group (11.15 MJ/kg, 14.52% CP), and (3) the high-energy and low-protein (HL) diet group (11.57 MJ/kg, 14.52% CP) (Table 1). All diets (Table 1) were formulated to meet the NY/T33-2004 requirements for hens [21], except for ME and CP in the 3 LP diets. The energy-to-protein ratios of the CK, LL, NL, and HL diets were 0.67, 0.74, 0.77, and 0.80, respectively.

**Table 1:** Composition and chemical analysis of the basic diets (air-dried basis)

Item	CK	LL	NL	HL
Ingredient, %				
Corn	61.60	62.87	66.95	64.43
Soybean meal	24.70	17.80	18.96	19.46
Wheat bran	2.00	7.40	2.00	2.00
Calcium hydrogen phosphate	0.80	0.65	0.83	0.86
Soybean oil	0.60	-	0.17	2.20
Limestone powder	9.30	9.48	9.35	9.32
Lysine	-	0.17	0.15	0.14
Methionine	-	0.01	0.03	0.03
Threonine	-	0.11	0.09	0.09
Tryptophan	-	0.01	0.03	0.02
Arginine	-	0.22	0.17	0.17
Leucine	-	0.17	0.14	0.15
Isoleucine	-	0.11	0.11	0.10
Cysteine	-	-	0.02	0.03
Premix^1^	1.00	1.00	1.00	1.00
Total	100.00	100.00	100.00	100.00
Nutrition level				
ME, MJ/kg	11.15	10.73	11.15	11.57
crude protein, %	16.59	14.50	14.52	14.52
Ca, %	3.85	3.85	3.85	3.85
Total P, %	0.55	0.53	0.54	0.54
Lysine, %	0.82	0.84	0.83	0.83
Methionine, %	0.26	0.25	0.27	0.27
Threonine, %	0.62	0.64	0.63	0.63
Tryptophan, %	0.19	0.17	0.19	0.18
Arginine, %	1.10	1.14	1.09	1.10
Leucine, %	1.42	1.42	1.42	1.42
Isoleucine, %	0.66	0.66	0.66	0.66
Cysteine, %	0.29	0.27	0.28	0.29

^1^Supplying per kilogram of diet: VA 8,000–10,000 IU, VD_3_ 2,200–5,000 IU, VE 13 IU, VK_3_ 1.4–4.8 mg, VB_1_ 1.8 mg, VB_2_ 3.0 mg, VB_6_ 2.0 mg, VB_12_ 0.01 mg, nicotinamide 20 mg, D-calcium pantothenate 10 mg, folic acid 0.55 mg, D-biotin 0.15 mg, choline 380 mg, Fe 60 mg, Cu 8 mg, Mn 60 mg, Zn 60 mg, I 0.35 mg, Se 0.12–0.48 mg, Ca 60–180 mg

Laying hens were placed in the cage (H 0.40 m × W 0.39 m × D 0.40 m) equipped with a nipple drinker and an exterior feed. Chickens were raised in an enclosed, ventilated, conventional house under 16/8 h light/dark conditions, 20–25 °C and 55% relative humidity. According to the management procedure for breeding Peking Pink laying hens (Yukou Poultry Co., Ltd., Beijing, China), every bird was provided with 112 g diet/d and had free access to water. The experiment lasted 77 d, including a 7-d acclimation period and a 70-d experimental period. At the end of this experiment, 12 eggs from each group were obtained to measure egg quality. One chicken from each replicate was sacrificed by exsanguination. The liver and cecal digesta were harvested and stored at −80 °C for further analysis.

### Production performance and egg quality measurement

The final body weights of laying hens were recorded before sample collection. Egg number and weight were recorded daily. Feed conversion efficiency was calculated as grams of feed intake. Yolk weight, yolk%, egg protein quality (Haugh unit), and albumen height were determined using an egg quality analyzer (Robotmation EMT-7300, Tokyo, Japan). Eggshell thickness (air cell, equator, and sharp end), egg length, and egg width were determined using a vernier caliper. Eggshell strength was determined using an egg force analyzer (Robotmation Model-III, Tokyo, Japan).

### Measurement of lipid metabolism indices in liver

Triglyceride (TG), total cholesterol (TCH), low-density lipoprotein cholesterol (LDL-C), lipase (LIP), hepatic lipase (HLP), and lipoprotein lipase (LPL) were detected using TG, TCH, and LDL-C kits (Nanjing Jiancheng Bioengineering Institute, Nanjing, China). High-density lipoprotein (HDL) cholesterol (HDL-C) was detected by HDL-C kits (Suzhou Comin Biotechnology Co. Ltd., Suzhou, China), following the manufacturer’s protocols. The liver enzyme activities of fatty acid synthase (FAS) and acetyl-CoA carboxylase (ACC) were detected using ELISA kits (Nanjing Jiancheng Bioengineering Institute, Nanjing, China).

### Liver RNA isolation and transcriptome sequencing

According to the results of lipid metabolism indicators, the low energy level in the LP diet led to lipid metabolism disorders. Therefore, the CK and LL groups were selected for subsequent multi-omics studies. Total RNA from the livers of laying hens in CK and LL groups was extracted using the Trizol reagent kit (Invitrogen, Carlsbad, CA, USA) according to the manufacturer’s protocol. After quality check by agarose gel electrophoresis, mRNA was enriched with oligo(dT) beads. Paired-end 150-bp sequencing libraries were constructed using the TruSeq™ RNA Sample Preparation Kit (Illumina, San Diego, CA, USA) and sequenced using an Illumina NovaSeq 6000 platform by Shanghai BIOZERON Co., Ltd. (Shanghai, China). Quality control for raw reads was executed by fastp software with the following parameters: unknown nucleotides < 10% and Q20 > 50% [22]. Bowtie2 was used for mapping reads to an rRNA database [23], and rRNA mapped reads were removed. Reference genome mapping and gene expression calculation were performed by HISAT2 [24] and RESM [25], respectively. Differentially expressed genes (DEGs) were identified by DESeq2 software [26] with the threshold values: fold change > 2 and FDR-adjusted *P*-value < 0.05. Thereafter, Kyoto Encyclopedia of Genes and Genomes (KEGG) annotations of DEGs were obtained from the reference genome and enrichment analysis was performed by clusterProfiler 4.0 [27]. Finally, the protein–protein interaction (PPI) network of DEGs was generated using String v10 [28], and the results were visualized using Cytoscape v3.7.1 [29].

### Non-targeted metabolomics for cecum contents

To extract the metabolites, 100 mg of cecal content of each hen from the CK and LL groups were snap-frozen in liquid nitrogen immediately after dissection. Then, samples were homogenized with 200 μL of water and five ceramic beads. Subsequently, the homogenate was resuspended with 800 μL pre-chilled methanol/acetonitrile (1:1, v:v). The mixture was centrifuged for 15 min (14,000 × *g* at 4 °C) and the supernatant was dried in a vacuum centrifuge. The supernatant was re-dissolved in 100 μL acetonitrile/water (1:1, v:v) and injected onto an ultra-high-pressure liquid chromatography (UHPLC) system (1290 Infinity LC, Agilent Technologies) coupled to a quadrupole time-of-flight mass spectrometer (AB Sciex TripleTOF 6600) to separate and detect metabolites. The UHPLC-MS/MS procedure was previously described in detail [30]. The peaks of metabolites were selected using XCMS software based on the raw MS data with the following parameters: centWave *m/z* = 10 ppm, peakwidth = c (10, 60), prefilter = c (10, 100). For peak grouping, bw = 5, mzwid = 0.025, minfrac = 0.5. Compound identification was performed by comparing accurate *m/z* value (< 10 ppm) and MS/MS spectra with an in-house database established with authentic standards. Differentially abundant metabolites (DAMs) were identified based on the following parameters: *P* < 0.05 (Student’s *t*-test), VIP > 1, and fold change > 1.5.

### Cecum microbiome sequencing

Microbial DNA was extracted from cecal contents using the QIAamp DNA Stool Mini Kit (QIAGEN, CA, USA) according to the manufacturer’s instructions. The quality of extracted DNA was checked by 1.5% agarose gel electrophoresis and a NanoPhotometer (IMPLEN, Germany). The V3–V4 region of the bacterial 16S rRNA gene was amplified by the primers 341F and 806R with an adapter sequence and barcode at the end of the reverse primer [31]. PCR amplification and library construction were performed as previously described [32]. Libraries were sequenced using an Illumina NovaSeq 6000 platform with a 250-bp paired-end strategy at BIOZERON Biotech. Co., Ltd. (Shanghai, China). After sequencing, the obtained raw reads were appointed to the samples based on their unique barcode. Then, reads with average Phred scores lower than 20, that contained ambiguous bases or had more than eight homopolymer runs, that had mismatches in the primers, and/or that had sequence lengths shorter than 250 bp were removed [33]. Paired reads were assembled, chimeras were eliminated, and clean data were clustered to the amplicon sequence variants (ASVs) using the DADA2 plugin unit in the QIIME2 program [34]. Singletons (where the number of a specific ASV was one) were removed and all remained ASVs were assigned to a taxon using the SILVA database (Release 138) [35]. The function of the cecal microbiome based on KEGG pathways was further predicted with PICRUSt2 software using the normalized ASV abundance table [36].

### Statistical analysis

All statistical analyses were performed in R v4.2.2 [37] and results were visualized by the “ggplot2” [38] and "pheatmap" packages [39]. First, the indices of production performance, egg quality, and liver lipid metabolism were compared by Tukey’s HSD test using the “multcomp” package. Subsequently, 4 alpha diversity indices of cecum microbiome, related to different facets, including richness (Chao1), evolution (Faith’s phylogenetic diversity, Faith_pd), diversity (Shannon), and evenness (Pielou’s evenness index, Pielou_J), were calculated by the “vegan” package. Differences in alpha diversity indices, relative abundances of dominant gut bacteria, and functional pathways of the cecal microbiome between the CK and LL groups were tested by the Student’s *t*-test. Meanwhile, the unweighted and weighted UniFrac distances between the cecal microbiome were obtained by the “GUniFrac” package [40]. Principal coordinate analysis (PCoA) and the adonis test based on these distances were executed with the “ape” and “vegan” packages to assess the effects of LP diets on the composition and function of cecal microbiome in laying hens. In addition, specificity and occupancy of each ASV in samples from the CK and LL groups were calculated and projected onto a SPEC-OCCU plot to explore specialists [41]. ASVs with specificity and occupancy values greater or equal to 0.7 were identified as specialists, indicating that they were specific to a specific diet.

To visualize the co-occurrence network of the cecal microbiome, Spearman’s rank correlations among all ASVs of samples from the CK and LL groups were calculated with the “WGCNA” package. A correlation between two genes was considered statistically robust if |correlation coefficient| > 0.8 and *P* < 0.01. Network graphs were visualized using the Gephi interactive platform and the topological parameters of networks were calculated with the “igraph” package [42]. Robustness and vulnerability of co-occurrence networks were calculated to evaluate the stability of the cecal microbiome according to a previous study [43]. The topological roles of individual ASVs in networks were evaluated by the threshold values of Pi (measuring how well a node was connected to nodes in different modules) and Zi (measuring how well a node was connected to other nodes in its own module) to identify potential key taxa [44]. Finally, correlations among the lipid metabolism indices in liver, DEGs in liver, DAMs in cecum, and key functional terms and bacteria in the cecal microbiome were analyzed by the Pearson correlation method with the “psych” package to explore the potential interactions in the gut-liver axis.

## Results

### Laying performance

As shown in Table 2, dietary treatment did not have a significant effect on laying rate and feed efficiency. The final body weight and average daily gain were significantly higher (*P* < 0.05) in the HL group than in the CK and LL groups. The egg weight was significantly higher (*P* < 0.05) in the HL group than in the LL group.

**Table 2:** Effects of different energy levels in LP diet on the laying performance of aged laying hens

Item	CK^1^	Dietary energy level in LP diet^2^			SEM^3^	*P*-value
		LL	NL	HL		
Initial body weight, g	1,904.04	1,897.83	1,905.50	1,905.33	1.945	0.324
Final body weight, g	2,039.17^bc^	1,987.00^c^	2,056.83^ab^	2,107.83^a^	11.263	< 0.001
Average daily gain, g/d	1.67^bc^	1.27^c^	2.16^ab^	2.89^a^	0.154	< 0.001
Egg weight, g	62.35^ab^	61.46^b^	62.52^ab^	63.25^a^	0.222	0.028
Laying rate, %	82.05	86.46	82.68	80.94	0.010	0.256
Feed efficiency, g/g	2.26	2.23	2.27	2.29	0.025	0.820

^1^The positive control group (CK): Normal-energy and normal-protein diet

^2^The experimental group (IELP) included the low-energy and low-protein diet group (LL), the normal-energy and low-protein diet group (NL), and the high-energy and low-protein diet group (HL)

^3^SEM = standard error of the mean

### Egg quality

The effects of different energy levels in the LP diets on egg quality are shown in Table 3. No significant differences in the egg shape index, eggshell thickness, eggshell strength, albumen height, yolk weight, and yolk ratio were observed among different treatment groups. In contrast, the Haugh units were significantly lower (*P* < 0.05) in the HL group than in the CK group.

**Table 3:** Effects of different energy levels in LP diet on the egg quality of aged laying hens

Item	CK^1^	Dietary energy level in LP diet^2^			SEM^3^	*P*-value
		LL	NL	HL		
Average egg weight, g	62.77	59.79	62.35	61.97	0.654	0.398
Egg shape index, %	1.29	1.27	1.30	1.29	0.004	0.090
Eggshell thickness, mm	0.37	0.36	0.34	0.35	0.0180	0.116
Eggshell strength, N	3.92	4.13	3.67	3.97	0.108	0.545
Albumen height, mm	6.84	6.28	6.62	5.97	0.210	0.501
Haugh units	81.73^a^	81.39^ab^	79.02^ab^	71.92^b^	1.402	0.034
Yolk weight, g	17.45	17.45	16.74	17.03	0.155	0.297
Yolk ratio	0.28	0.30	0.27	0.28	0.005	0.262

^1^The positive control group (CK): Normal-energy and normal-protein diet

^2^The experimental group (IELP) included the low-energy and low-protein diet group (LL), the normal-energy and low-protein diet group (NL), and the high-energy and low-protein diet group (HL)

^3^SEM = standard error of the mean

### Liver fat accumulation

**Fig. 1:**
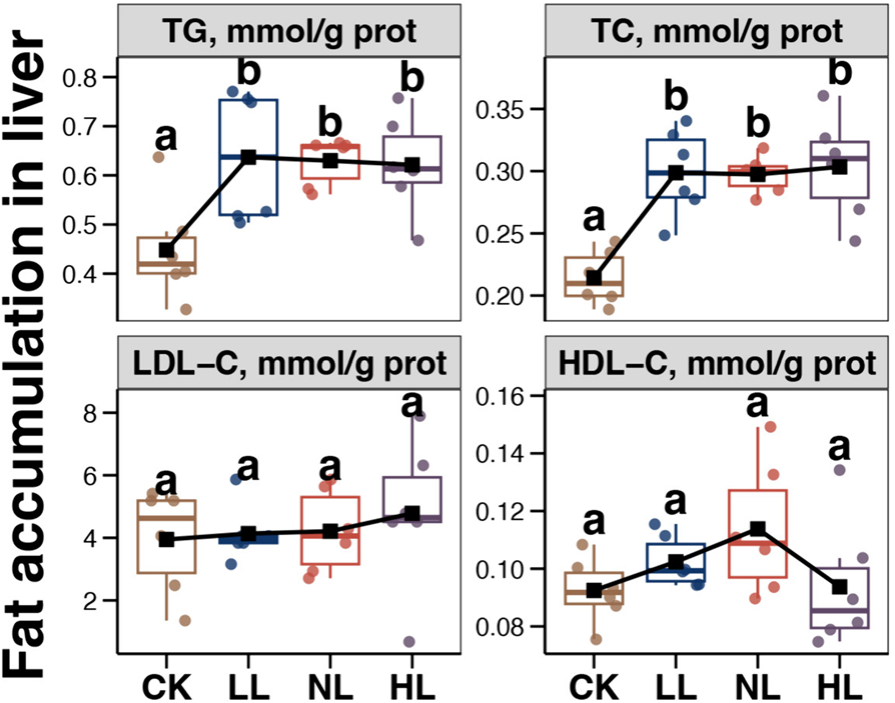
Differences of fat level indice in the liver of aged laying hens among different groups. Different lowercase letters in each box of the same sub-figure represent significant differences among aged laying hens from different groups (Tukey’s HSD test, *P* < 0.05)

The effects of different energy levels on TG, TC, LDL-C, and HDL-C in the laying hens fed the LP diet are shown in Fig. 1. Compared with the CK group, TG and TC levels were higher in the hens fed LP diets (LL, NL, and HL groups). In contrast, there were no significant differences in LDL-C and HDL-C levels among the 4 groups.

### Lipid metabolism-related enzymes in the liver

**Fig. 2:**
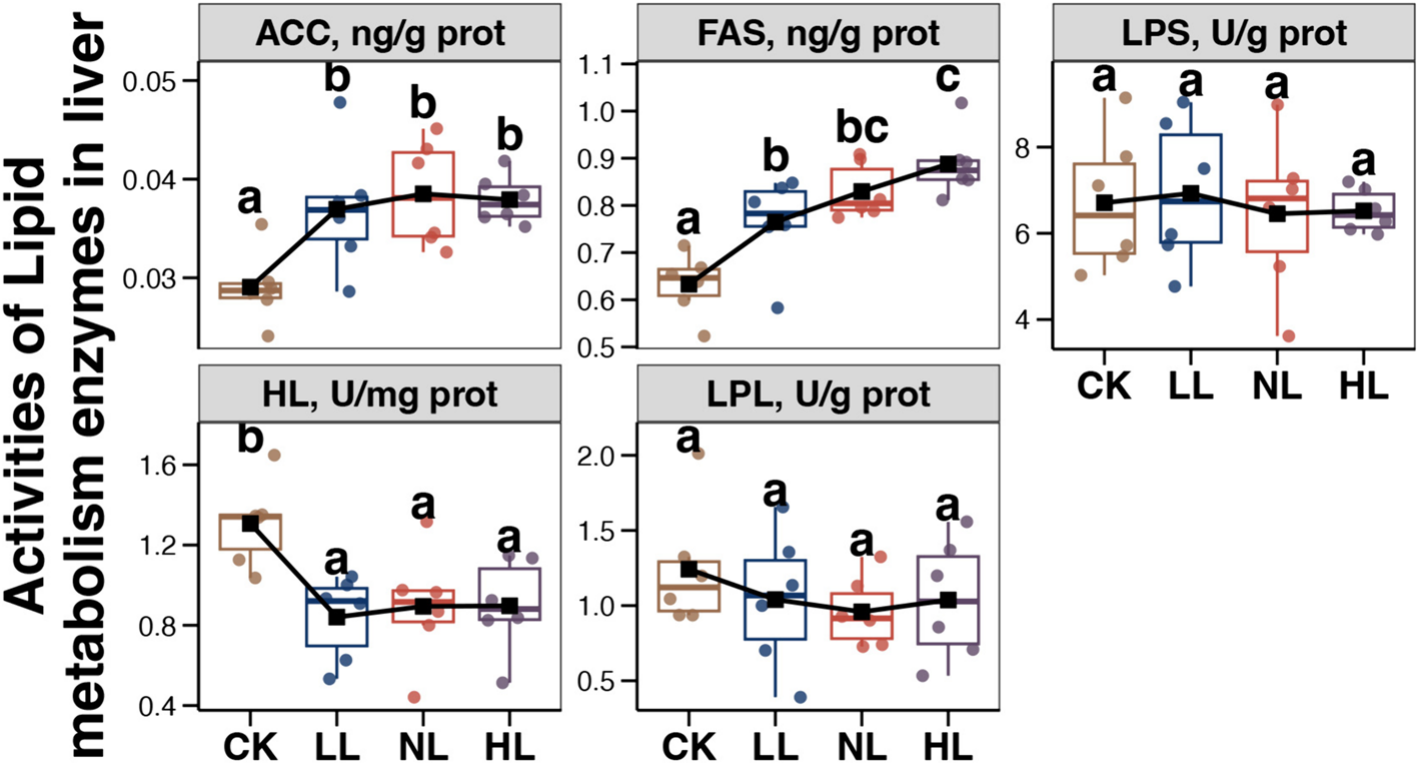
Variations in the activities of lipid metabolic enzymes in the liver of aged laying hens among different groups. Different lowercase letters in each box of the same sub-figure represent significant differences among aged laying hens from different groups (Tukey’s HSD test, *P* < 0.05)

The effects of different energy levels in LP diets on the activities of liver lipid metabolism-related enzymes are shown in Fig. 2. Compared with the CK group, ACC and FAS levels were significantly increased (*P* < 0.05), but that of HLP was decreased (*P* < 0.05) in the livers of laying hens fed LP diets (LL, NL, and HL groups). There were no significant differences in LPS activity among the 4 groups.

### Gene expression in liver

Based on the above results, the low energy level in the LP diet led to liver lipid metabolism disorders in laying hens. Therefore, the CK and LL groups were selected to study the relationship between gut microbes and liver lipid metabolism using a multi-omics strategy.

**Fig. 3:**
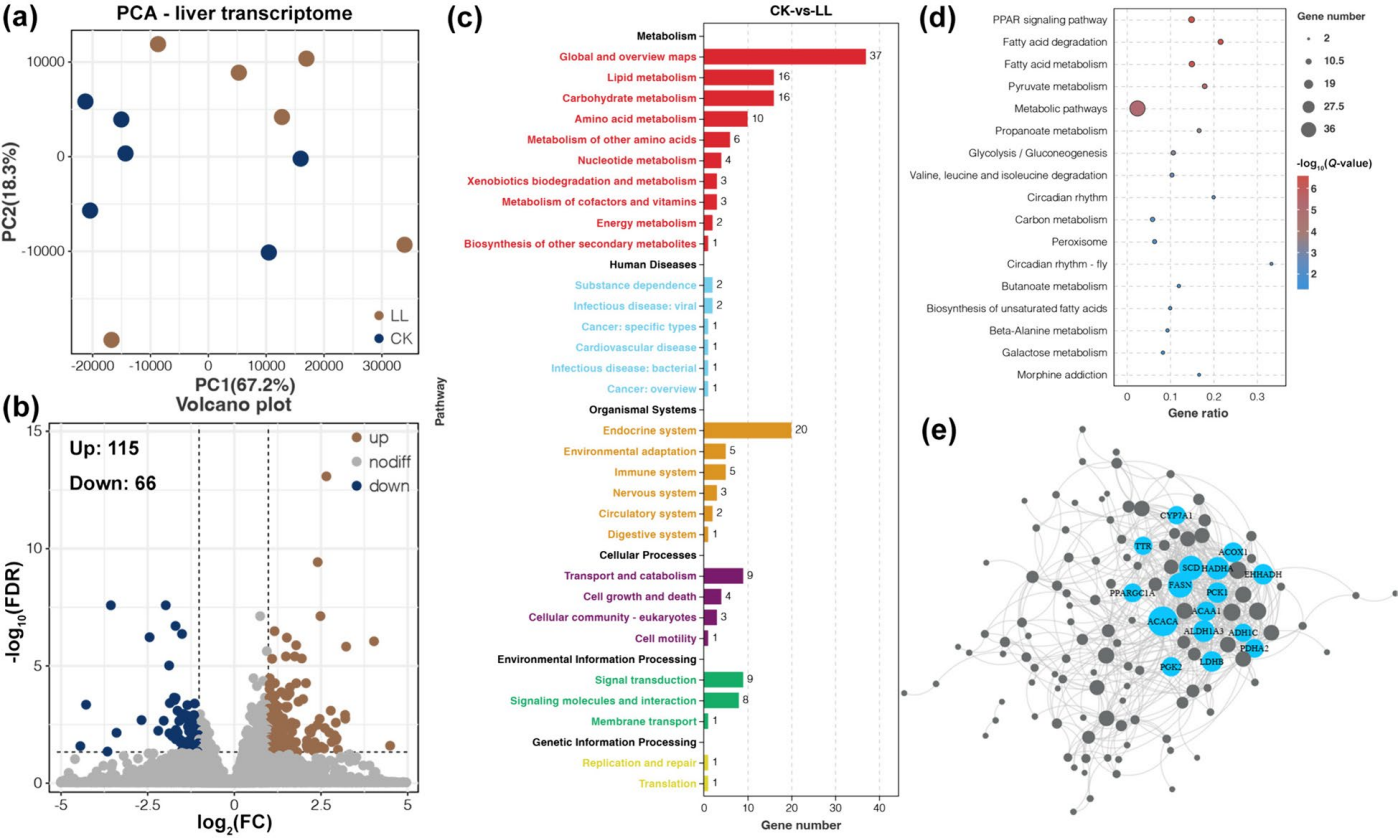
Liver transcriptome of aged laying hens fed an LP diet. **a** PCA exhibiting the variations in the gene expression profiles. **b** Volcano plot showing the results of DEG identification. **c** KEGG annotation of DEGs. **d** KEGG enrichment analysis of DEGs. **e** The PPI network of DEGs

For both the CK and LL groups, six libraries were constructed for RNA-Seq analysis. From the library, 42,423,838 to 51,104,686 sequenced reads were generated with a low-quality ratio below 1% and approximately 1% rRNA reads (Table S1). Approximately 95% of the clean reads were mapped to the reference genome, with an exon rate > 85% and 17,007 genes (Table S2). Principal components analysis (PCA) was performed according to gene expression levels, and the results showed distinct gene expression profiles between the CK and LL samples (Fig. 3a). Subsequently, 181 DEGs were identified from the transcriptomic data, with 115 upregulated and 66 downregulated genes in the livers of the LL group compared to those in the CK group (Fig. 3b). The DEGs were mainly related to lipid, carbohydrate, and amino acid metabolism (Fig. 3c). In addition, some genes related to the endocrine system, transport, and signal transduction were differentially expressed between the CK and LL groups (Fig. 3c). Furthermore, the KEGG enrichment analysis indicated that the LL diet influenced the liver functions of the laying hens related to lipid metabolism (fatty acid metabolism and degradation), energy metabolism (pyruvate metabolism, glycolysis/gluconeogenesis, and galactose metabolism), amino acid metabolism (valine, leucine, and isoleucine degradation, and beta-alanine metabolism), circadian rhythm, and PPAR signaling pathway (Fig. 3d). Finally, the PPI network of DEGs revealed some potential key genes that were influenced by the LP diet, including genes related to fatty acid beta-oxidation (*ACOX1*, *HADHA*, *EHHADH*, and *ACAA1*), fatty acid biosynthesis (*SCD*, *FASN*, and *ACACA*), lipid transport (*TTR*, *ALDH1A3*, and *ADH1C*), and energy metabolism (*CYP7A1*, *PCK1*, *PPARGC1A*, *PGK2*, *PDHA2*, and *LDHB*) (Fig. 3e).

### Metabolites in cecum

**Fig. 4:**
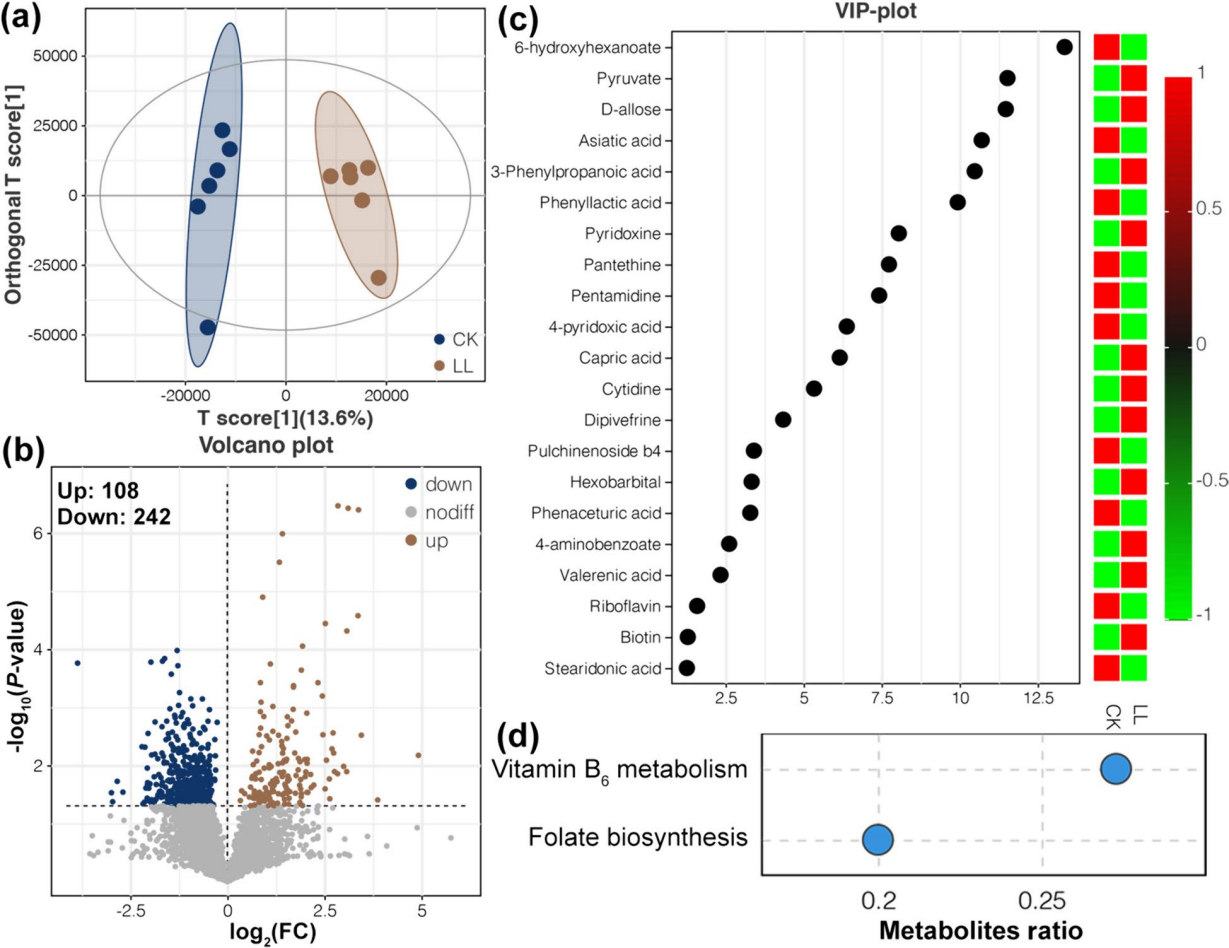
Cecal metabolites of aged laying hens in the CK and LL groups. **a** OPLS-DA revealing the variations in cecal metabolites. **b** Volcano plot showing the results of DAM identification. **c** VIP plot exhibiting the DAMs with the strongest variation between the CK and LL groups. **d** KEGG pathway enrichment analysis of DAMs

A total of 5,149 metabolites were detected in CK and LL samples using UPLC-MS/MS metabolic analysis. Orthogonal partial least square discriminant analysis (OPLS-DA) revealed that cecal metabolites in hens fed the same diet were clustered together and away from others (Fig. 4a), suggesting obvious effects of the LL diet on the cecal metabolism of the laying hens. In total, 350 DAMs were identified among the cecal metabolites between the LL and CK groups, with 108 upregulated and 242 downregulated DAMs in the LL group (Fig. 4b). Cecal metabolites that were most significantly impacted by the LL diet included riboflavin (vitamin B_2_), pantethine (a derivative of vitamin B_5_), pyridoxine (vitamin B_6_), 4-pyridoxic acid, cytidine, biotin, 6-hydroxyhexanoate, pyruvate, and asiatic acid (Fig. 4c). Moreover, the vitamin B_6_ metabolism and folate biosynthesis pathways were found to be enriched in the cecum of laying hens fed an LL diet (Fig. 4d).

### Cecal microbiome

**Fig. 5:**
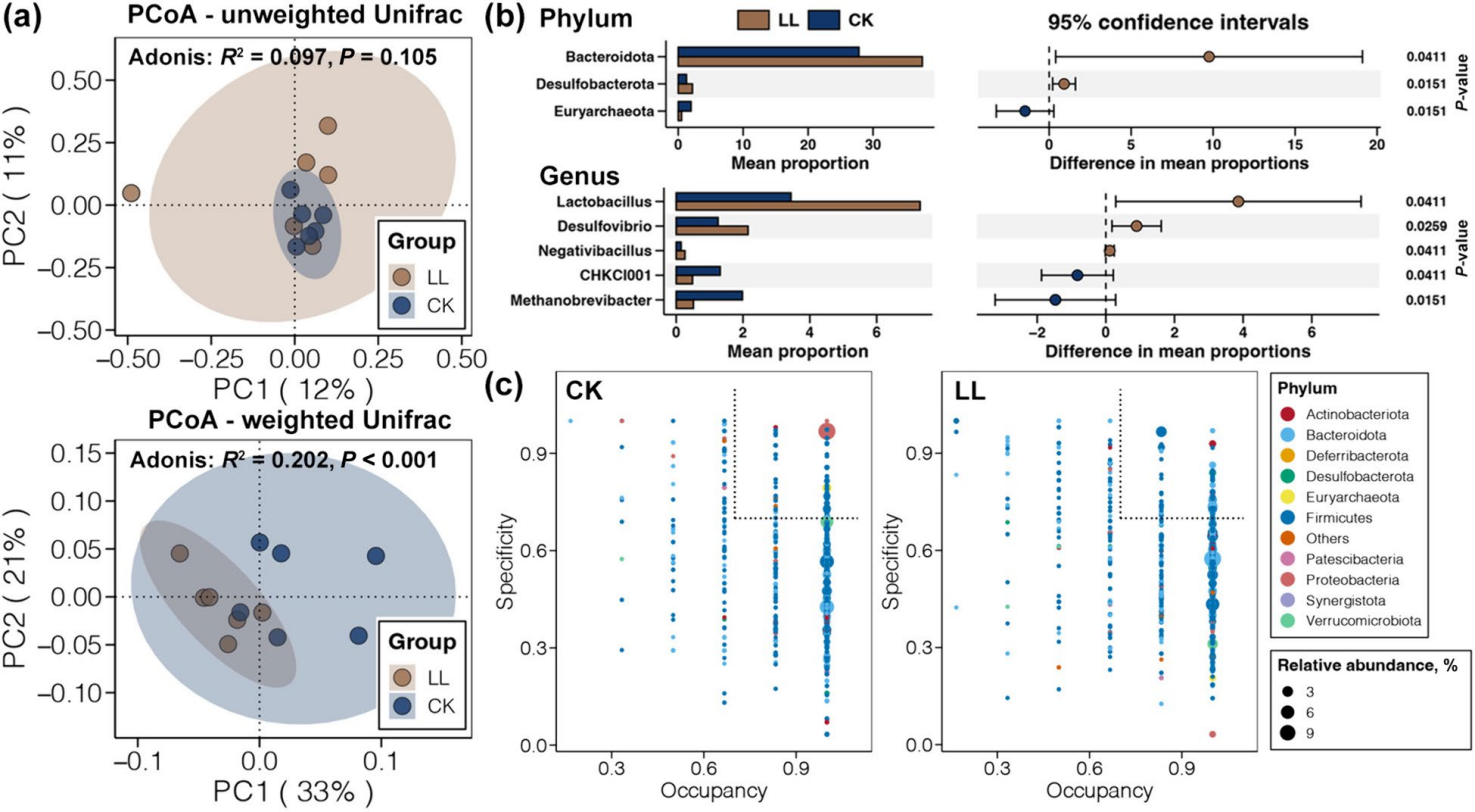
Variations in the composition of the cecal microbiome induced by the LP diet. **a** PCoA and the adonis test based on the unweighted and weighted UniFrac distances revealing the differences in cecal microbiome composition between the CK and LL groups. **b** Bacterial phyla and genera with significant variation in relative abundance in the cecum between the CK and LL groups (Student’s *t*-test, *P* < 0.05). **c** SPEC-OCCU plots showing ASVs in the cecal microbiome of aged laying hens fed a normal or LP diet

On average, 129,625 reads of the 16S rRNA V3–V4 region were obtained for the cecal microbiome of CK and LL samples (Table S2). After quality control, 93,573 to 107,196 high-quality reads were retained per sample, with an effective ratio of 78.39% (Table S3). These reads were clustered into 6,804 ASVs with an average of 1,164 ASVs per sample, which were annotated to 25 phyla, 43 classes, 100 orders, 132 families, 168 genera, and 107 species (Table S3). All ASVs were successfully annotated to a bacterial phylum, but only 36.78% ASVs were assigned to a taxon at the genus level (Fig. S1a). Generally, the numbers of obtained bacteria at all taxonomic levels were higher in LL samples compared to CK samples (Table S3). However, changes in the alpha diversity of cecal microbiome induced by the LL diet were not significant (Student’s *t*-test, *P* < 0.05, Fig. S1b). PCoA and the adonis test based on the unweighted UniFrac distance also showed insignificant variations in the compositions of the cecum microbiome between the CK and LL groups (*P* > 0.05, Fig. 5a), but significant changes in the cecal microbiome compositions were revealed by the PCoA and adonis test based on the weighted UniFrac distance (*P* < 0.05, Fig. 5a). The unweighted UniFrac distance only considers the composition of the species without taking into account their relative abundance; in contrast, the weighted UniFrac distance considers not only the composition of species but also their relative abundance [45]. Our findings indicate that the changes in the cecal microbiome induced by the LL diet are mainly variations in the relative abundance of major bacteria.

Firmicutes and Bacteroidota dominated in the cecal microbiomes of laying hens, followed by Proteobacteria and Verrucomicrobiota (Fig. S2). Bacteroidota and Desulfobacterota were more abundant in the cecum of laying hens fed the LL diet, while the relative abundance of Euryarchaeota was decreased (Student’s *t*-test, *P* < 0.05, Fig. 5b). At the genus level, *Rikenellaceae RC9 gut group* was the most abundant, followed by *Bacteroides*, *Ruminococcus torques group*, *Escherichia-Shigella*, and *Lactobacillus* (Fig. S3). The relative abundances of *Lactobacillus*, *Desulfovibrio*, and *Negativibacillus* were increased by the LL diet, while *CHKCI001* and *Methanobrevibacter* were eliminated (Student’s *t*-test, *P* < 0.05, Fig. 5b). Moreover, SPEC-OCCU plots were applied to explore potential specialists in the cecum microbiome of laying hens fed the LP diet (Fig. 5c). A total of 57 and 55 ASVs were recognized as potential specialists for the normal and LP diets, respectively. Most of them were members of Firmicutes (46 and 35 for CK and LL groups, respectively). More importantly, the number of specialists belonging to the Bacteroidota was only 4 in the CK group, but it was 17 in the LL group.

**Fig. 6:**
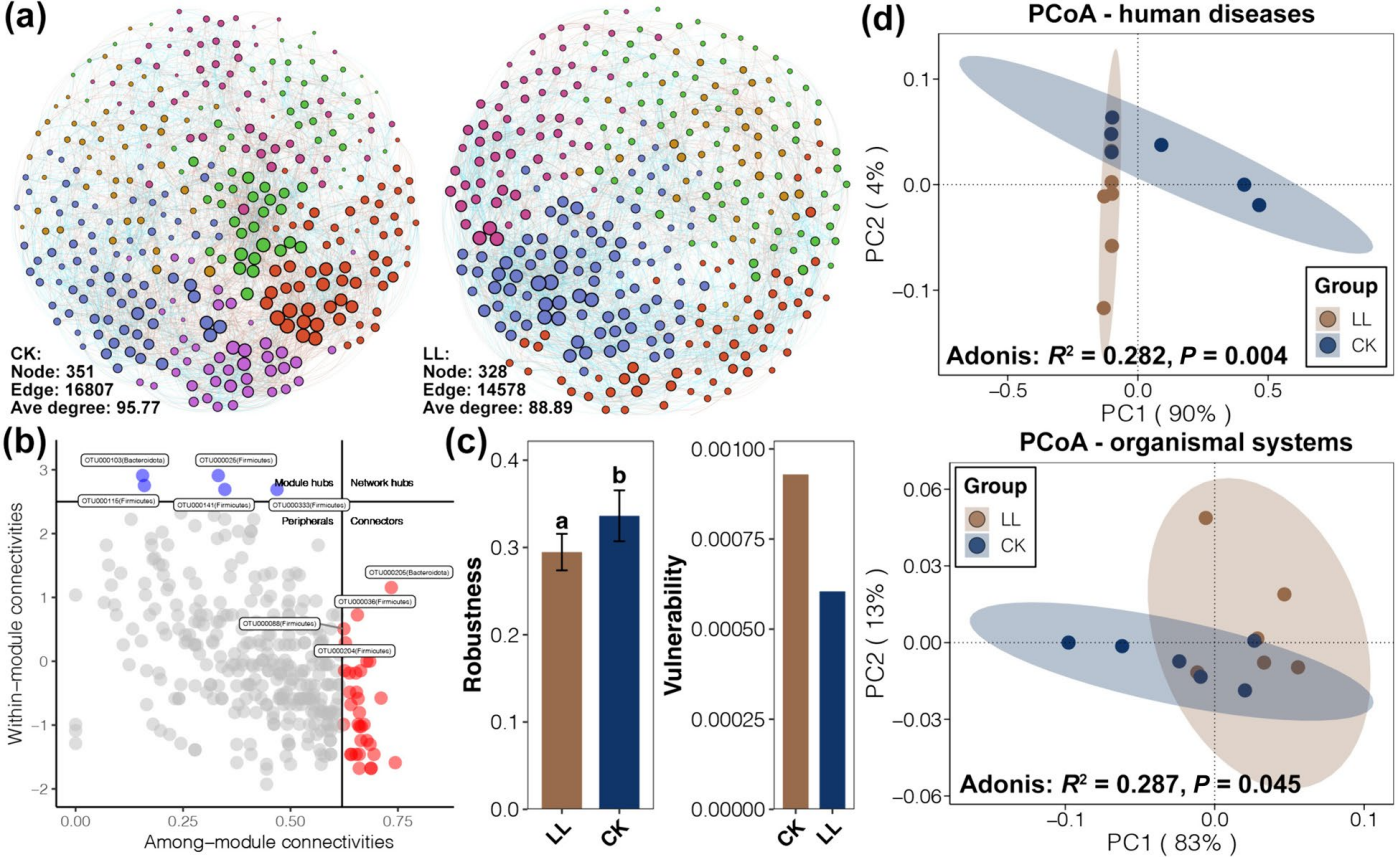
Co-occurrence network and predicted function of the cecal microbiome. **a** Co-occurrence networks of the cecal microbiome from the CK and LL samples. Nodes belonging to different modules are labeled in different colors. **b** Zi-Pi plot showing the distribution of bacterial ASVs based on their topological roles. **c** Robustness and vulnerability of networks of the LL and CK groups. Different lowercase letters above the bars of the robustness plot represent a significant difference between the CK and LL groups (Student’s *t*-test, *P* < 0.05). **d** PCoA and adonis test based on the Bray–Curtis distance revealing the differences in cecal microbiome functions related to human disease and organismal systems between the CK and LL groups

Co-occurrence networks of the cecal microbiome of laying hens fed a normal or LL diet were further constructed (Fig. 6a). The co-occurrence pattern in cecal microbiome was slightly simple for laying hens fed by the LL diet according to the topological parameters of networks (lower number of nodes, number of edges, and degrees). Meanwhile, five ASVs were recognized as module hubs of the co-occurrence network, including 4 Firmicutes and one Bacteroidota (Fig. 6b). Lower robustness and higher vulnerability were found in network of LL samples compared to CK individuals (Fig. 6c), suggesting weak stability of the cecal microbiome in the laying hens fed the LL diet. In addition, the predicted functions of cecal microbiota were compared between the LL and CK groups. Although changes in the whole cecal microbiome functions were insignificant (adonis test, *P* > 0.05, Fig. S4), obvious differences in the functions related to human disease and organismal systems were found between the cecal microbiome of the LL and CK groups (adonis test, *P* < 0.05, Fig. 6d and Fig. S5). Moreover, two metabolism functions, lipid metabolism and secondary bile acid biosynthesis, were found to be enriched in the cecal microbiome of laying hens fed the LL diet (Student’s *t*-test, *P* < 0.05, Fig. S6). In contrast, functions including C5-branched dibasic acid metabolism, nitrotoluene degradation, and riboflavin metabolism in the cecal microbiome of laying hens were weakened by the LL diet (Student’s *t*-test, *P* < 0.05, Fig. S6).

### Roles of the gut-liver axis

**Fig. 7:**
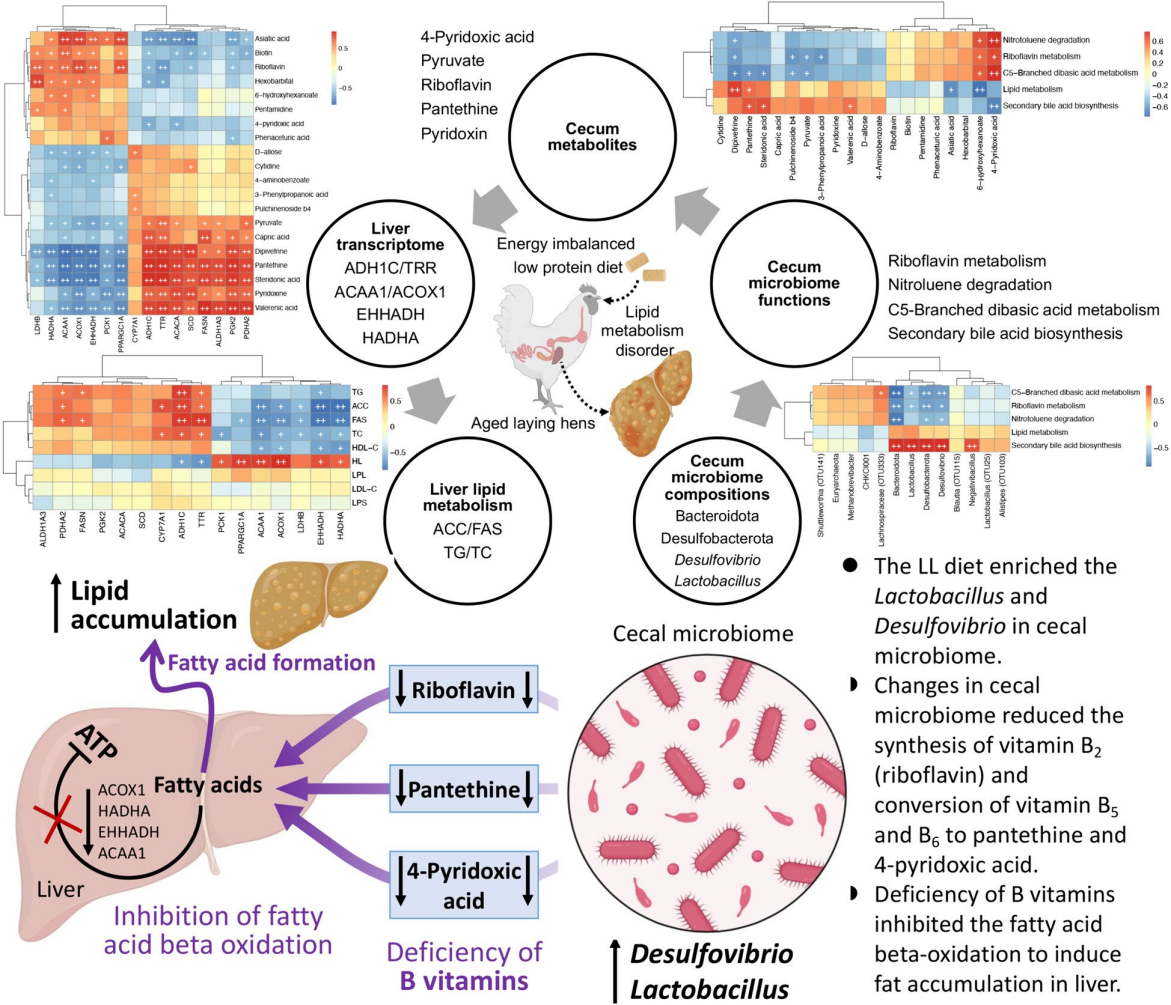
Correlation analyses among liver lipid metabolism indices, liver DEGs, cecum DAMs, different cecal microbiome functions, and different cecal bacteria, and the conceptual frameworks of deduced mechanisms of lipid metabolism disorder in aged laying hens fed the LL diet

To explore the underlying mechanisms of liver lipid metabolism disorder in the laying hens fed LL diets, correlation analyses were performed between any two indices of liver lipid metabolism, liver DEGs, cecal DAMs, different cecal microbiome functions, and different cecal bacteria (Fig. 7). Based on the significant relationships, potential keystones related to lipid metabolism disorder were observed, including 4 liver lipid metabolism indices, 6 liver genes, 5 cecal metabolites, 4 cecal microbial functions, and 4 cecal bacteria. According to these functions and significant correlations, we deduced the underlying mechanism for lipid metabolism disorder in the laying hens fed the LL diet. LL dietary treatment enriched *Desulfovibrio* and *Lactobacillus* in the cecum of the laying hens, which inhibited the biosynthesis of vitamin B_2_ (riboflavin), pantethine (a derivative of vitamin B_5_), and 4-pyridoxic acid (the end product of vitamin B_6_ catabolism). The deficiency of B vitamins restrained the expression of genes related to the beta-oxidation of fatty acids (*ACOX1*, *HADHA*, *EHHADH*, and *ACAA1*) in liver, resulting in the accumulation of lipid in the liver.

## Discussion

Liver lipid metabolism disorders often occur in late-phase laying hens after experiencing peak production [46]. We explored mechanisms underlying the harmful effects of the energy/protein imbalanced LP diets on laying hens to provide recommendations for improvement. Previous studies have indicated that the production performance and egg quality of laying hens fed the energy/protein imbalanced LP diet were decreased [13, 47]. Our results are consistent with these studies, showing an adverse effect of the HL diet on laying rate and egg quality. As the late-phase laying hens, the increased weight and the amount of abdominal fat may reduce production performance [48]. In the present study, the final body and egg weight was markedly increased in the HL group which was harmful to the late-phase laying hens according to the management procedure for breeding Peking Pink laying hens (Yukou Poultry Co., Ltd., Beijing, China). More importantly, our findings demonstrated that the energy/protein imbalanced LP diet disrupted lipid metabolism and resulted in fat accumulation in the livers of the late-phase laying hens, which is consistent with previous findings [49]. The principal cause of lipid metabolism disorders in laying hens is thought to be excessive calorie intake, which leads to an incorrect energy/protein balance in the diet [50, 51]. In this study, the energy-to-protein ratios in the LP groups (LL, NL, and HL groups) were much higher than that in the CK group.

The expression levels of some genes involved in fatty acid synthesis were upregulated in the livers of the late-phase laying hens fed LP diets (Fig. 3). *ACACA* encodes a biotin-containing enzyme that catalyzes the carboxylation of acetyl-CoA to malonyl-CoA, which is the rate-limiting step in fatty acid synthesis [52]. *FASN* encodes a multifunctional protein that catalyzes the synthesis of palmitate from acetyl-CoA and malonyl-CoA into long-chain saturated fatty acids in the presence of NADPH [53]. The effect of these genes on the energy/protein imbalanced LP diet was further confirmed by the results of enzyme activities (Fig. 2). In contrast, genes involved in the beta-oxidation of fatty acids were downregulated in the livers of the late-phase laying hens fed an energy/protein imbalanced LP diet (Fig. 3). *ACOX1* encodes the first enzyme in the fatty acid beta-oxidation pathway that catalyzes the desaturation of acyl-CoAs to 2-trans-enoyl-CoAs [54]. *HADHA* encodes the alpha subunit of the mitochondrial trifunctional protein, which catalyzes the last 3 steps of the mitochondrial beta-oxidation of long-chain fatty acids [55]. *EHHADH* and *ACAA1* encode 2 of the 4 enzymes involved in the peroxisomal beta-oxidation pathway [56, 57]. These findings suggest that fat accumulation in the liver of late-phase laying hens fed an energy/protein imbalanced LP diet was attributable to both promotion of fatty acid biosynthesis and the suppression of fatty acid utilization.

The composition and function of the gut microbiota are closely related to host health, and diet is an important factor in regulating the homeostasis of gut microbiota [58]. Several studies have revealed the potential negative effects of an energy/protein imbalanced LP diet on the gut microbiota of animals [59]. Our results revealed that two bacterial genera, *Lactobacillus* and *Desulfovibrio*, might be key cecal microbes that respond to the energy/protein imbalanced LP diet, both of which were enriched in hens fed LL diets (Fig. 5b). A study on broiler chickens showed that reduced intake of crude protein increased the abundance of *Lactobacillaceae* in the cecum over time [60]. In addition, Zhong et al. [61] indicated that a high-protein diet could reduce gut *Lactobacillus*, which was consistent with the results of this study. Furthermore, Raman et al. [62] and Nobili et al. [63] reported that gut *Lactobacillus* were markedly increased in humans with lipid metabolic disorders of the liver. In contrast, another cecal bacterium enriched by the energy/protein imbalanced LP diet, *Desulfovibrio*, has been associated with several diseases, including inflammatory bowel disease, bacteremia, and Parkinson’s disease [64]. Moreover, research has shown that *Desulfovibrio* dysregulation is associated with fatty acid levels and modulation of *Desulfovibrio* may be a potential strategy for the treatment of lipid metabolism disorders [65]. Our results highlight the possible roles of *Lactobacillus* and *Desulfovibrio* in the cecal microbiome of laying hens during the development of lipid metabolism disorders.

In our study, we observed that some cecal metabolites which lower liver fatty acid decomposition were significantly decreased in late-phase laying hens fed the LL diets, such as riboflavin (vitamin B_2_) and pantethine (a derivative of vitamin B_5_) (Fig. 4c), whose deficiency has been demonstrated to impair lipid metabolism by depressing fatty acid beta-oxidation [66–68]. Subsequently, association analyses based on multi-omics data were performed to explore the underlying relationships between variations in the cecal microbiome and lipid metabolism disorders in liver with a deficiency of riboflavin and pantethine in laying hens fed an energy/protein imbalanced LP diet. These findings revealed potential avenues for further research on dietary interventions, microbiota modulation, and the development of targeted treatments to support the liver health of poultry.

Riboflavin, also known as vitamin B_2_, is a key vitamin that plays an important role in breaking down nutrients in food to produce energy [69]. Animals cannot synthesize vitamin B_2_, which is primarily sourced from food or is synthesized by the gut microbiota [70]. The role of vitamin B_2_ in functional rescue of mitochondrial beta-oxidation flavoenzymes for fatty acid degradation has also been proven [71]. Our results showed that riboflavin metabolism was inhibited in the cecal microbiome of the late-phase laying hens fed an LL diet. Significant correlations were observed among the enriched cecal bacteria (*Lactobacillus* and *Desulfovibrio*), decreased riboflavin metabolism in the cecal microbiome, downregulated cecal riboflavin, and downregulated liver genes related to beta-oxidation of fatty acids (*ACOX1*, *HADHA*, *EHHADH*, and *ACAA1*) (Fig. 7). Moreover, vitamin B_6_ (pyridoxine), a B-complex vitamin, was significantly enriched and 4-pyridoxic acid (the primary catabolic product of vitamin B_6_) was scarce in the ceca of the late-phase laying hens fed an LL diet. Decreased levels of 4-pyridoxic acid are associated with impaired fatty acid beta-oxidation in poultry [72, 73]. In addition to its metabolic role, vitamin B_2_ enables the conversion of vitamin B_6_ into its active form [74]. Previous studies have also found that riboflavin is an important determinant of vitamin B_6_ status in healthy adults [75, 76]. Significant correlations between the levels of vitamins B_2_, B_6_, and 4-pyridoxic acid were observed (Fig. 7). These results indicate that the metabolic rate of vitamin B_6_ is inhibited in laying hens fed the LL diet due to deficiencies in vitamin B_2_, which leads to the accumulation of vitamin B_6_, but insufficient 4-pyridoxic acid in the cecum. Taken together, we deduced that variations in the cecal microbiome of the late-phase laying hens induced by the energy/protein imbalanced LL diet led to vitamin B_2_ deficiency, which further inhibited the beta-oxidation of fatty acids in the liver and resulted in lipid metabolism disorders.

Pantethine, another downregulated cecal metabolite, is the active form of pantothenic acid (vitamin B_5_), which is an essential component of coenzyme A (CoA) [67, 77, 78]. CoA is involved in the transport of fatty acids to the mitochondria, where fatty acid beta-oxidation occurs [79]. CoA is also the only source of the phosphopantetheine prosthetic group for enzymes that shuttle intermediates between the active sites of enzymes involved in fatty acid metabolism [80]. In addition, CoA is a source of the 4'-phosphopantetheinyl prosthetic group present in the acyl carrier protein, which is required for fatty acid synthesis in biological systems [81]. Many studies have demonstrated that pantethine has anticatabolic properties in fatty acid synthesis and stimulates fatty acid oxidation [82, 83]. Pantetheine has been used in humans as a lipid-lowering drug and dietary pantethine has also been shown to improve liver lipogenesis in laying hens [84, 85]. In our study, the decreased level of pantethine in the cecum of the late-phase laying hens fed an LL diet was also significantly correlated with the downregulated expression of genes related to fatty acid beta-oxidation and enriched key cecal bacteria (Fig. 7). These associations indicate that the lipid metabolism disorder caused by the LP diet in late-phase laying hens may also be related to the pantethine deficiency induced by variations in the cecal microbiome.

## Conclusions

The energy/protein imbalanced LP diet significantly altered fat accumulation in the livers of late-phase laying hens. The inhibition of beta-oxidation and promotion of fatty acid biosynthesis in the liver of the late-phase laying hens directly contributed to the fat accumulation induced by the energy/protein imbalanced LP diet. The energy/protein imbalanced LP diet influenced the cecal microbiome, resulting in enriched *Lactobacillus* and *Desulfovibrio* genera. Moreover, our results revealed the deficiencies of vitamins B_2_ and pantethine in late-phase laying hens fed an energy/protein imbalanced LP diet owing to changes in the cecal microbiome. This may explain the weak fatty acid beta-oxidation ability and strengthened fatty acid synthesis in the liver. These results provide insights into mechanisms underlying liver lipid metabolism disorders and their association with the gut-liver axis in the late-phase laying hens fed LP diets. Based on these results, we propose that supplementing vitamin B_2_ and pantethine may effectively prevent lipid metabolism disorders in the late-phase laying hens fed an LP diet.

### Supplementary Information

**Additional file** **1:** **Table S1**. Statistics of transcriptome sequencing for liver of aged laying hens. **Table S2**. Statistics of cecal microbiome sequencing of aged laying hens. **Table S3**. Numbers of taxa detected in cecal microbiome of aged laying hens. **Fig. S1**. (a) Ratio of successful annotation of ASVs in cecal microbiome of aged laying hens. (b) Variations in the alpha diversity of cecal microbiome of aged laying hens among different treatments. **Fig. S2**. Relative abundance of major bacterial phyla in cecum of aged laying hens. **Fig. S3**. Relative abundance of top 10 bacterial genera in cecal microbiome of aged laying hens fed by the LP and normal diets. **Fig. S4**. PCoA and adonis test based on the Bary-Curtis distance revealing the changes in cecal microbiome function between the CK and LL groups. **Fig. S5**. PCoA and adonis test based on the Bary-Curtis distance revealing the changes in cecal microbiome function related to metabolism, cellular processes, environmental information processing, and genetic information processing between the CK and LL groups. **Fig. S6**. Bacterial functions with significant variations in relative abundance in the cecum between the CK and LL groups (Student’s *t*-test, *P* < 0.05).

## Data Availability

The raw sequencing data have been deposited in the China National GeneBank Sequence Archive (CNSA) of the China National GeneBank DataBase (CNGBdb) with the accession number CNP0005028 and CNP0005031.

## Abbreviations

ACAA1: Acetyl-CoA acyltransferase 1
ACACA: Acetyl-CoA carboxylase alpha
ACC: Acetyl-CoA carboxylase
ACOX1: Acyl-CoA oxidase 1
ADH1C: Alcohol dehydrogenase 1C
ALDH1A3: Aldehyde dehydrogenase 1 family member A3
ASVs: Amplicon sequence variants
CK: Positive control
CoA: Coenzyme A
CYP7A1: Cytochrome P450 family 7 subfamily A member
DAMs: Differentially abundant metabolites
DEGs: Differentially expressed genes
EHHADH: Enoyl-CoA hydratase and 3-hydroxyacyl CoA dehydrogenase
Faith_pd: Faith’s phylogenetic diversity
FAS: Fatty acid synthase
FASN: Fatty acid synthase
KEGG: Kyoto Encyclopedia of Genes and Genomes
HADHA: Hydroxyacyl-CoA dehydrogenase trifunctional multienzyme complex subunit alpha
HDL: High-density lipoprotein
HDL-C: High-density lipoprotein cholesterol
HL: High-energy and low-protein diet
HLP: Hepatic lipase
LDHB: Lactate dehydrogenase B
LDL-C: Low-density lipoprotein cholesterol
LIP: Lipase
LL: Low-energy and low-protein diet
LP: Low-protein
LPL: Lipoprotein lipase
NL: Normal-energy and low protein diet
OPLS-DA: Orthogonal partial least square discriminant analysis
PCA: Principal components analysis
PCoA: Principal coordinate analysis
PCK1: Phosphoenolpyruvate carboxykinase 1
PDHA2: Pyruvate dehydrogenase E1 subunit alpha 2
PGK2: Phosphoglycerate kinase 2
Pielou_J: Peilou’s evenness index
PPI: Protein–protein interaction
PPARGC1A: Peroxisome proliferator activated receptor gamma coactivator 1 alpha
SCD: Stearoyl-CoA desaturase
TCH: Total cholesterol
TG: Triglyceride
TTR: Transthyretin
