# Supplementary material for: Effects of different energy levels in low-protein diet on liver lipid metabolism in the late-phase laying hens through the gut-liver axis
Source: J Anim Sci Biotechnol. 2024 Jul 11;15:98. doi: 10.1186/s40104-024-01055-y (PMC11238517; doi:10.1186/s40104-024-01055-y)
Supplement: Supplementary file 1 — Additional file 1: Table S1. Statistics of transcriptome sequencing for liver of aged laying hens. Table S2. Statistics of cecal microbiome sequencing of aged laying hens. Table S3. Numbers of taxa detected in cecal microbiome of aged laying hens. Fig. S1. (a) Ratio of successful annotation of ASVs in cecal microbiome of aged laying hens. (b) Variations in the alpha diversity of cecal microbiome of aged laying hens among different treatments. Fig. S2. Relative abundance of major bacterial phyla in cecum of aged laying hens. Fig. S3. Relative abundance of top 10 bacterial genera in cecal microbiome of aged laying hens fed by the LP and normal diets. Fig. S4. PCoA and adonis test based on the Bary-Curtis distance revealing the changes in cecal microbiome function between the CK and LL groups. Fig. S5. PCoA and adonis test based on the Bary-Curtis distance revealing the changes in cecal microbiome function related to metabolism, cellular processes, environmental information processing, and genetic information processing between the CK and LL groups. Fig. S6. Bacterial functions with significant variations in relative abundance in the cecum between the CK and LL groups (Student’s t-test, P < 0.05). [file 40104_2024_1055_MOESM1_ESM.docx]

**Table S1** Statistics of transcriptome sequencing for liver of aged laying hens

| **Sample** | **Raw reads** | **Clean reads** | **rRNA reads** | **Ref mapped** | **exon rate** |
| --- | --- | --- | --- | --- | --- |
| LL1 | 43,769,120 | 99.62% | 0.41% | 95.72% | 86.19% |
| LL2 | 51,104,686 | 99.59% | 0.42% | 95.44% | 87.52% |
| LL3 | 42,792,442 | 99.19% | 0.92% | 94.21% | 88.11% |
| LL4 | 49,042,024 | 99.54% | 1.05% | 95.25% | 86.26% |
| LL5 | 47,317,806 | 99.55% | 0.88% | 95.70% | 86.61% |
| LL6 | 50,685,382 | 99.56% | 0.58% | 95.27% | 86.73% |
| CK1 | 44,971,994 | 99.27% | 0.63% | 95.05% | 86.86% |
| CK2 | 44,698,680 | 99.55% | 0.88% | 95.21% | 86.95% |
| CK3 | 45,432,300 | 99.57% | 0.61% | 95.25% | 87.63% |
| CK4 | 42,765,990 | 99.31% | 0.51% | 95.51% | 87.00% |
| CK5 | 42,423,838 | 99.29% | 1.58% | 95.47% | 87.03% |
| CK6 | 47,016,096 | 99.53% | 0.63% | 95.15% | 87.79% |

**Table S2** Statistics of cecal microbiome sequencing of aged laying hens

| **Sample ID** | **Raw reads** | **Clean reads** | **Raw tags** | **Clean tags** | **Chimera** | **Effective tags** | **Effective ratio, %** |
| --- | --- | --- | --- | --- | --- | --- | --- |
| LL-1 | 134,269 | 133,274 | 121,278 | 118,315 | 13,205 | 105,110 | 78.28 |
| LL-2 | 133,772 | 132,632 | 120,495 | 117,702 | 13,275 | 104,427 | 78.06 |
| LL-3 | 136,224 | 135,063 | 121,874 | 119,565 | 12,294 | 107,271 | 78.75 |
| LL-4 | 121,989 | 121,073 | 110,032 | 105,258 | 11,685 | 93,573 | 76.71 |
| LL-5 | 135,396 | 134,109 | 120,517 | 117,675 | 12,610 | 105,065 | 77.6 |
| LL-6 | 123,919 | 123,713 | 118,210 | 116,879 | 14,459 | 102,420 | 82.65 |
| MM-1 | 122,087 | 120,952 | 108,634 | 105,610 | 11,487 | 94,123 | 77.1 |
| MM-2 | 126,594 | 125,499 | 114,043 | 111,654 | 12,785 | 98,869 | 78.1 |
| MM-3 | 122,219 | 121,253 | 109,913 | 107,393 | 11,551 | 95,842 | 78.42 |
| MM-4 | 128,798 | 127,741 | 116,081 | 113,300 | 11,584 | 101,716 | 78.97 |
| MM-5 | 136,562 | 135,504 | 121,972 | 118,199 | 11,003 | 107,196 | 78.5 |
| MM-6 | 133,667 | 132,579 | 120,331 | 115,914 | 12,306 | 103,608 | 77.51 |

**Table S3** Numbers of taxa detected in cecal microbiome of aged laying hens

| **Taxonomy** | **LL** | **CK** | **Total** |
| --- | --- | --- | --- |
| Phylum | 24 | 16 | 25 |
| Class | 41 | 23 | 43 |
| Order | 96 | 47 | 100 |
| Family | 126 | 69 | 132 |
| Genus | 159 | 117 | 168 |
| Species | 89 | 93 | 107 |


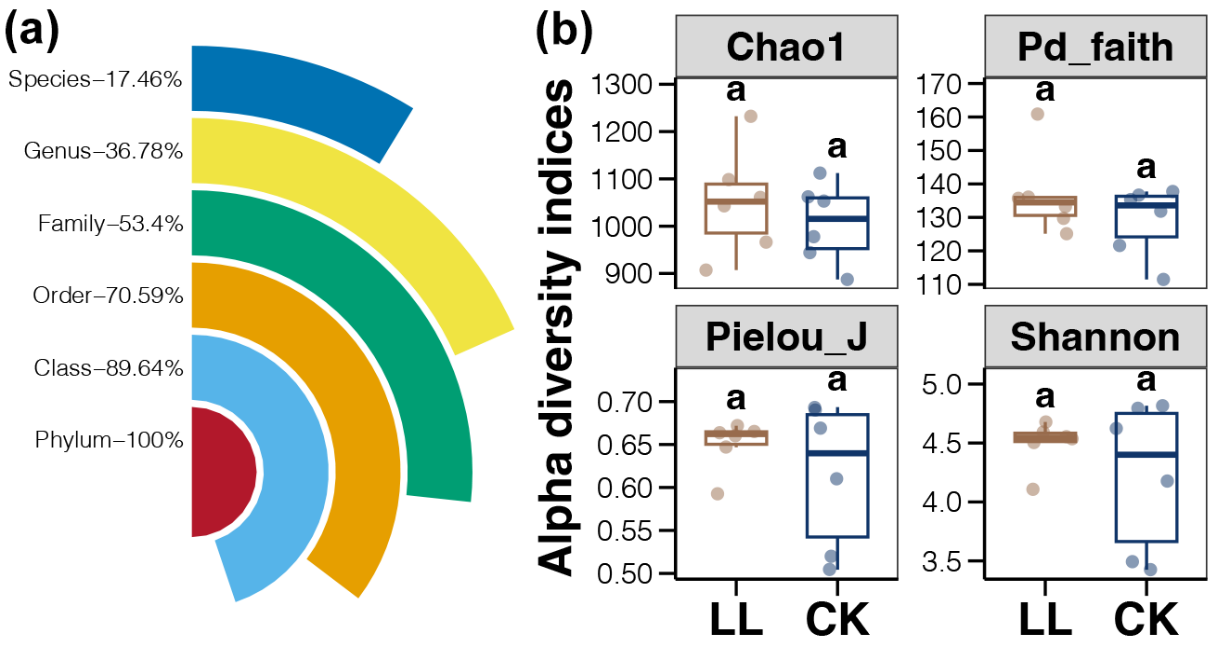


**Fig. S1** (a) Ratio of successful annotation of ASVs in cecal microbiome of aged laying hens. (b) Variations in the alpha diversity of cecal microbiome of aged laying hens among different treatments. Different lowercases letters in each box of the same sub-figure represent significant differences among aged laying hens from different treatments (Tukey’s HSD test, *P* < 0.05)

**
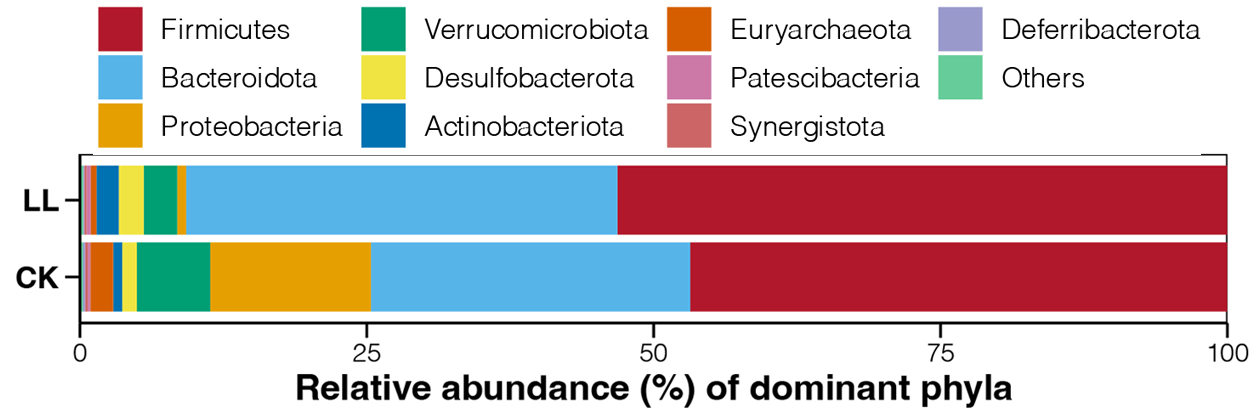
**

**Fig. S2** Relative abundance of major bacterial phyla in cecum of aged laying hens

**Fig. S3** Relative abundance of top 10 bacterial genera in cecal microbiome of aged laying hens fed by the LP and normal diets


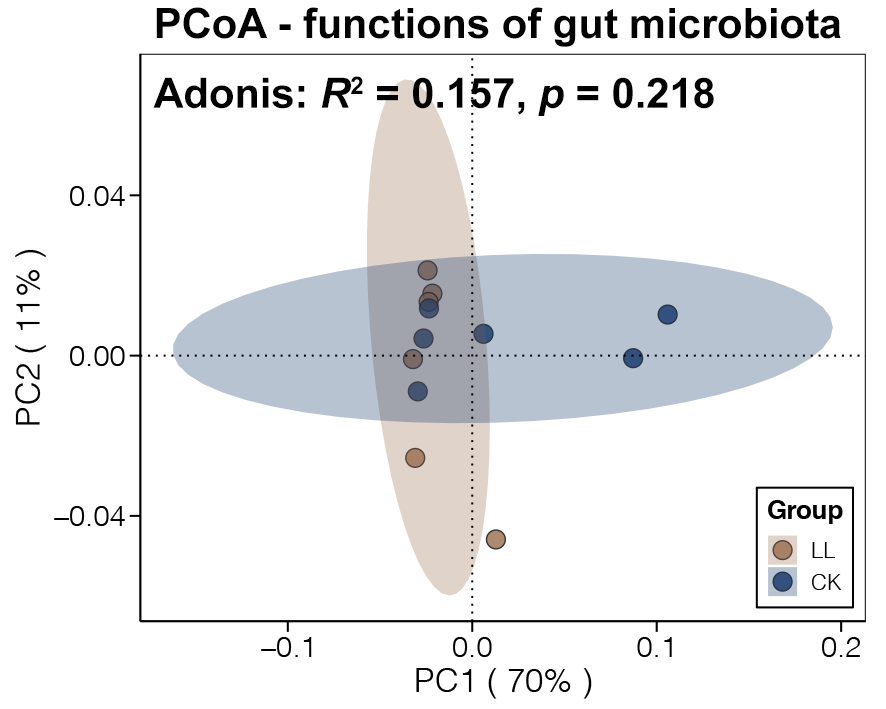


**Fig. S4** PCoA and adonis test based on the Bary-Curtis distance revealing the changes in cecal microbiome function between the CK and LL groups

**
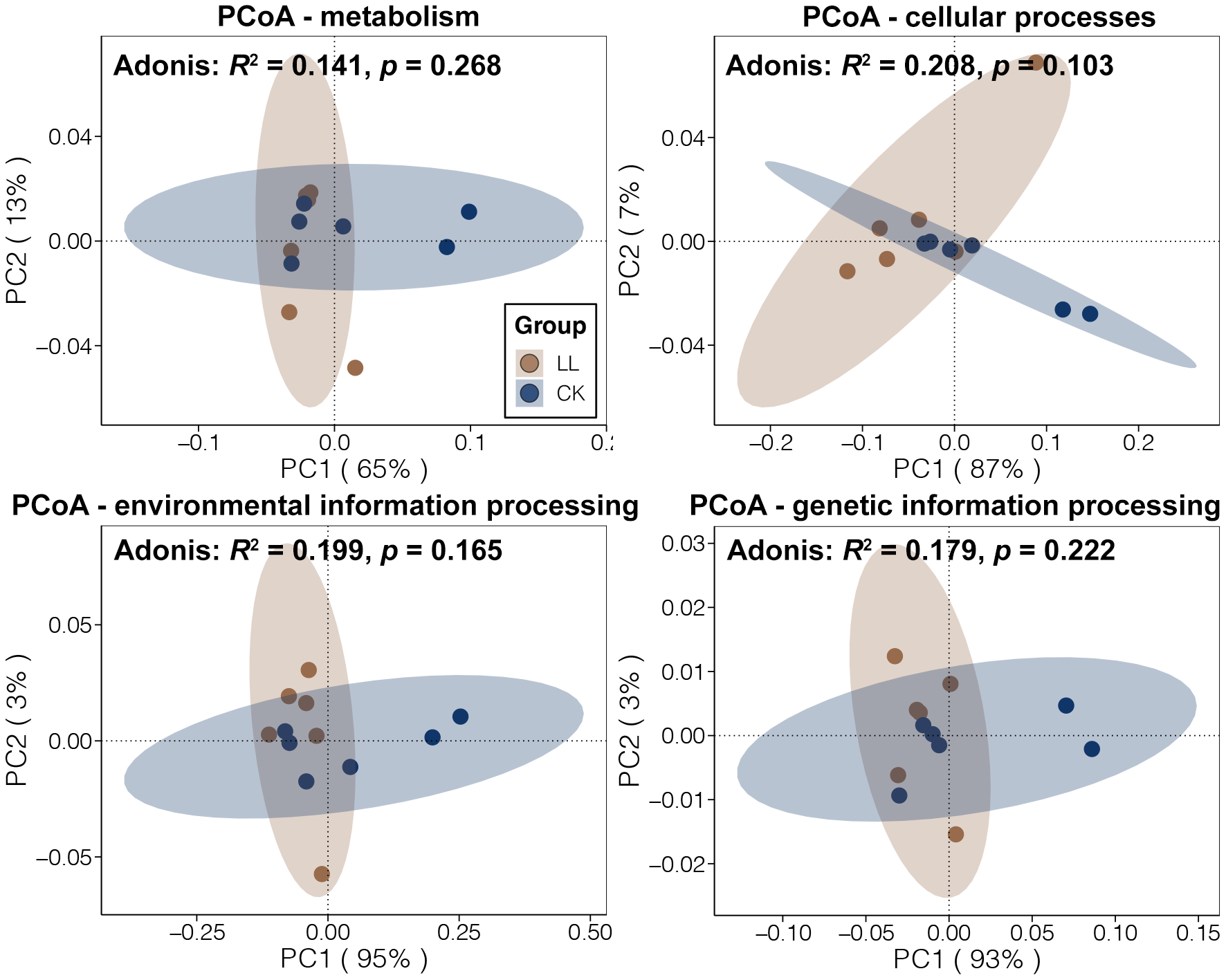
**

**Fig. S5** PCoA and adonis test based on the Bary-Curtis distance revealing the changes in cecal microbiome function related to metabolism, cellular processes, environmental information processing, and genetic information processing between the CK and LL groups

**
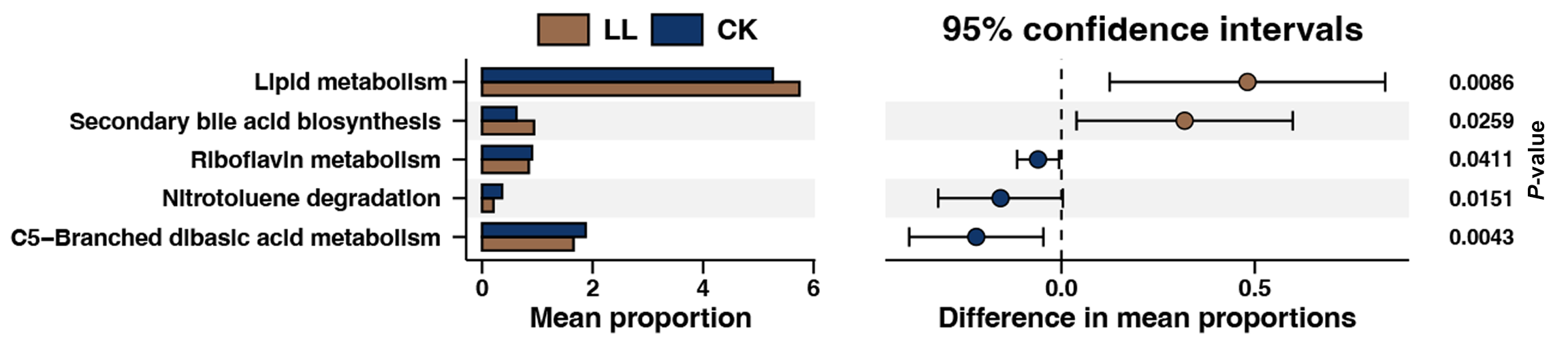
**

**Fig. S6** Bacterial functions with significant variations in relative abundance in the cecum between the CK and LL groups (Student's *t*-test, *P* < 0.05)
